# Odds ratios from logistic, geometric, Poisson, and negative binomial regression models

**DOI:** 10.1186/s12874-018-0568-9

**Published:** 2018-10-20

**Authors:** Christopher J. Sroka, Haikady N. Nagaraja

**Affiliations:** 1 Department of Economics, Applied Statistics, and International Business, New Mexico State University, MSC 3CQ, PO Box 30001, Las Cruces, NM, 88003-8001 USA; 2 Division of Biostatistics, The Ohio State University, 1841 Neil Avenue, Columbus, OH, 43210-1240 USA

**Keywords:** Binary data, Confidence intervals, Count data, Fisher information, Maximum likelihood

## Abstract

**Background:**

The odds ratio (OR) is used as an important metric of comparison of two or more groups in many biomedical applications when the data measure the presence or absence of an event or represent the frequency of its occurrence. In the latter case, researchers often dichotomize the count data into binary form and apply the well-known logistic regression technique to estimate the OR. In the process of dichotomizing the data, however, information is lost about the underlying counts which can reduce the precision of inferences on the OR.

**Methods:**

We propose analyzing the count data directly using regression models with the log odds link function. With this approach, the parameter estimates in the model have the exact same interpretation as in a logistic regression of the dichotomized data, yielding comparable estimates of the OR. We prove analytically, using the Fisher information matrix, that our approach produces more precise estimates of the OR than logistic regression of the dichotomized data. We also show the gains in precision using simulation studies and real-world datasets. We focus on three related distributions for count data: geometric, Poisson, and negative binomial.

**Results:**

In simulation studies, confidence intervals for the OR were 56–65% as wide (geometric model), 75–79% as wide (Poisson model), and 61–69% as wide (negative binomial model) as the corresponding interval from a logistic regression produced by dichotomizing the data. When we analyzed existing datasets using our approach, we found that confidence intervals for the OR could be up to 64% shorter (36% as wide) compared to if the data had been dichotomized and analyzed using logistic regression.

**Conclusions:**

More precise estimates of the OR can be obtained directly from the count data by using the log odds link function. This analytic approach is easy to implement in software packages that are capable of fitting generalized linear models or of maximizing user-defined likelihood functions.

**Electronic supplementary material:**

The online version of this article (10.1186/s12874-018-0568-9) contains supplementary material, which is available to authorized users.

## Background

Count data arise naturally in many biomedical applications. These data are often converted to binary values and commonly analyzed using logistic regression methods. Usually “failure” for a subject is defined as having a count of zero and “success” as having a positive count. If *p*_1_ is the probability of a positive count for the group with a risk factor of interest and *p*_2_ is the probability of a positive count for the group without the risk factor, then the two groups are typically compared using the odds ratio *OR*=[*p*_1_/(1−*p*_1_)]/[*p*_2_/(1−*p*_2_)].

Several examples of this dichotomization approach can be found. From a survey of dietary behaviors among Canadian youth, Vanderlee et al. [1] dichotomized the number of sugar-sweetened beverages consumed and compared the odds of consuming at least one beverage based on gender, age group, and physical activity. A similar approach was used to estimate the odds of one or more motor vehicle collisions by elderly drivers based on the frequency of falls [2] and the odds of one or more dental caries in children based on diet and obesity [3]. In some applications, the cutpoint of interest for the OR is not always at one. Van Strien et al. [4] defined frequent falls to be more than two in the past year, and dichotimized the count data around this value to calculate the odds of frequent falls associated with taking psychotropic medications. Duggal et al. [5] fit two separate models to examine the number of outpatient visits to Veterans Administration (VA) facilities. A logistic regression model was applied to the binary data of use or non-use of the VA facilities and a separate negative binomial (NB) model (with support of positive integers) was used for the count data of the frequency of visits among those who had at least one visit.

The loss of information from dichotomizing a continuous variable prior to analysis has been studied extensively. Suissa and Blais [6] examined dichotomizing continuous variables above and below thresholds of clinical interest (e.g., patients with cholesterol higher or lower than 240 mg/dl). They developed an approach for modeling the probability of being above the clinical threshold using a generalized linear model (GLM) framework and demonstrated the gains in information using this approach instead of a logistic regression with dichotomized values. Moser and Coombs [7] developed a method to estimate the OR directly from a continuous regression model by assuming that the errors follow a logistic distribution. Suissa [8] and Peacock et al. [9] applied the delta method to obtain an estimator and standard error for the OR (as well as other metrics of risk) using the sample mean and standard deviation from a normal distribution.

Research on the loss of information from dichotomizing count data is limited. Recently, Preisser et al. [10] examined the information loss in logistic regression when compared to a special two-part hurdle model with truncated Poisson or NB distribution for the observed count data. These models have separate parameters for altering the probability of zero counts and the mean of the truncated distribution used for positive counts. It is assumed the two parts of the model are related, with their link functions having common regression parameters involving covariates. The critical assumption is given in condition (6) of their paper. It assumes that the logit link function for the zero count and the log mean link function for the truncated count distribution are linearly related. The crucial difference between their hurdle model and the corresponding ordinary Poisson or NB count models is that their condition (6) is incompatible for these distributions. However it is compatible for the geometric model, in which case the link functions are identical.

## Methods

We propose analyzing the non-dichotomized data using count regression models with the log odds link function and demonstrate this method with the geometric, Poisson, and NB distributions. These three distributions are related to each other and can be used to model a wide range of overdispersion in the count data. We compare inference for the OR using our method to the logistic regression approach that dichotomizes the count data. Our focus is on analyses that compare the odds of a positive count between two groups (cutpoint at zero). The use of the log odds link function results in model parameters being compatible with the usual logistic regression model and enables us to directly compare the resulting covariate dependent OR estimates. In addition, the log odds link function is a function of the mean for all of these models and has the real line as the range space. Typically, count regression models use the so-called canonical link function, which differs for the three distributions examined here. As noted by McCullagh and Nelder ([11], p. 32), while the canonical link function has many desirable properties due to its special role in the exponential family of distributions, these properties do not justify its use when the application at hand suggests that a different link function is more appropriate. Here, the desire by researchers to report results in terms of the OR rather than other statistics implied by the canonical link (e.g., relative means) supports the use of alternative link functions. Cook ([12], p. 2091) lists some guiding principles on the choice of a link function.

### Fisher information in dichotomized count variables

Let *Y* be a random variable (r.v.) from a counting process with *p*= Pr(*Y*>0), 0<*p*<1, and support {0,1,…}. Define the binary r.v. *Z* to equal 0 if *Y*=0 and 1 if *Y*>0. The r.v. *Z* follows the Bernoulli distribution with mean *μ*=*p* and variance *v*(*μ*)=*p*(1−*p*)=*μ*(1−*μ*). Let *θ* denote the odds of a positive count; that is,

1 $$  \theta = \frac{\Pr(Y>0)}{\Pr(Y=0)} = \frac{\Pr(Z=1)}{\Pr(Z=0)} = \frac{p}{1-p}.  $$

Then the FI about *θ* in *Z* is

2 $$  FI(Z;\theta) = \frac{1}{\theta\left(1+\theta\right)^{2}}.  $$

We note that *Z* is not a sufficient statistic for the data generated by *Y*, and we expect *FI*(*Z*;*θ*) to be strictly less than the corresponding *FI*(*Y*;*θ*). We demonstrate this and quantify the magnitude of information loss for the three count models considered.

Logistic regression is often used to estimate the log odds from a vector of *n* independent Bernoulli r.v.’s **Z**=(*Z*_1_,…,*Z*_*n*_) conditional on a set of covariates. The means of these Bernoulli r.v.’s are related to the covariates through the link function *h*(*μ*)= log(*θ*)= log[*p*/(1−*p*)] that is linear in them as follows:

3 $$  \log \left[ \theta (\mathbf{x}_{i})\right] = \log\left[\frac{p(\mathbf{x}_{i})}{1 - p(\mathbf{x}_{i})}\right] = \mathbf{x}_{i}^{\prime} \boldsymbol{\beta} \quad i = 1, 2, \ldots, n,  $$

where $\mathbf {x}^{\prime }_{i} = (x_{i0} \equiv 1, x_{i1}, \ldots, x_{ik})$ is the 1×(*k*+1) vector corresponding to the *k* covariates associated with a single subject *i* and ***β***=(*β*_0_,*β*_1_,…,*β*_*k*_)^′^ is the (*k*+1)×1 vector of associated coefficients.

The FI in **Z** about an arbitrary *β*_*j*_ in the regression model is

4 $$\begin{array}{*{20}l}  FI(\mathbf{Z}; \beta_{j}) =& \sum\limits_{i=1}^{n} FI(Z_{i}; \theta) \left(\frac{\partial \theta}{\partial \beta_{j}}\right)^{2}  \\ =& \sum\limits_{i=1}^{n} p(\mathbf{x}_{i})[1-p(\mathbf{x}_{i})] x_{ij}^{2}. \end{array} $$

Using general results on GLMs [11], the (*j*,*m*)^th^ element of the FI matrix for ***β*** is given by

5 $$ -E\left[\frac{\partial^{2}\ell(\boldsymbol{\beta})}{\partial \beta_{j} \partial \beta_{m}}\right] = \sum\limits_{i=1}^{n} w(\mathbf{x}_{i}) x_{ij} x_{im}, \quad j, m= 1, \ldots, k,  $$

where *ℓ* is the log likelihood function and the weights are

6 $$ w(\mathbf{x}_{i})= \frac{1}{v(\mu(\mathbf{x}_{i}))\left[h^{\prime}(\mu(\mathbf{x}_{i}))\right]^{2}}, \quad 1 \leq i \leq n.  $$

For the logistic regression model, *v*(*μ*)=*μ*(1−*μ*)=1/*h*^′^(*μ*) and consequently *w*(**x**)=*p*(**x**)[1−*p*(**x**)].

In subsequent sections, we use the following result to compare the asymptotic variance of estimators of ***β*** by comparing the values of the weights *w*(**x**_*i*_) in the FI matrix.

**Result 1.** Let **I**_*C*_ be the FI matrix for ***β*** based on the count data **Y** with *w*(**x**_*i*_)=*w*_*C*_(*i*). Let **I**_*B*_ be the FI matrix for ***β*** based on the dichotomized count data **Z** with *w*(**x**_*i*_)=*w*_*B*_(*i*). If *w*^∗^(*i*)=*w*_*C*_(*i*)−*w*_*B*_(*i*)>0 for all *i*=1,…,*n*, then the asymptotic variance of the estimator of ***β*** is strictly less under the count model than under the logistic model.

To prove this result, define $\mathbf {X}^{\prime }_{(k+1) \times n}= (\mathbf {x}_{1}, \cdots, \mathbf {x}_{n})$, a matrix associated with the covariates, and $\mathbf {W}^{*}_{n\times n} = diag\{w^{*}(1), \ldots, w^{*}(n)\}$, a diagonal matrix with positive diagonal elements. Then it follows that **I**^∗^=**X**^′^**W**^∗^**X** is positive definite. Being FI matrices, **I**_*C*_ and **I**_*B*_ are already positive definite. Problem 9 in Rao ([13], p. 56) states that if **M**_2_ is a positive definite matrix and (**M**_1_−**M**_2_) is non-negative definite, then $\mathbf {M}^{-1}_{2} - \mathbf {M}^{-1}_{1}$ is also non-negative definite. By taking **M**_1_=**I**_*C*_ and **M**_2_=**I**_*B*_, it means the diagonal entries of the difference matrix $\mathbf {I}^{-1}_{B} - \mathbf {I}^{-1}_{C}$ are all positive. Thus we have proved the result.

### The geometric regression model

When the count variable *Y* has a geometric distribution with Pr(*Y*=0)=1−*p*, its probability mass function (pmf) is given by

7 $$ \Pr(Y = y) = p^{y} (1 - p), \quad y= 0, 1, 2, \ldots.  $$

The mean *μ* and variance functions for this geometric pmf are:

8 $$  \mu = \frac{p}{1 - p}, \qquad v(\mu) = \frac{p}{\left(1 - p \right)^{2}} = \mu (1 + \mu).  $$

The mean *μ* in (8) is nothing but the odds of a positive count (see (1)). Letting *θ*_*G*_ denote the odds from the geometric distribution and using (2), it can be easily shown that

9 $$  FI(Y;\theta_{G}) = \frac{1}{\theta_{G}(1+\theta_{G})} = (1 + \theta_{G}) FI(Z;\theta_{G}).  $$

As *Y* is non-degenerate, (1+*θ*_*G*_)>1 and there is more information in the count r.v. than in the dichotomized r.v. *Z*. Thus, the asymptotic relative efficiency (ARE) of the MLE of *θ*_*G*_ based on a random sample of size *n* from the geometric distribution when compared to the dichotomized data is (1+*θ*_*G*_). (See [14], for a definition of the ARE).

Now consider *n* independent r.v.’s **Y**=(*Y*_1_,…,*Y*_*n*_) from the geometric distribution and the corresponding dichotomized variables **Z**=(*Z*_1_,…,*Z*_*n*_). The log link function *h*(*μ*)= log(*μ*) is commonly used in count models [15]. In the case of the geometric distribution, this link function is identical to log[*p*/(1−*p*)], the same link function commonly used for models of the dichotomized data, and the covariates affect the parameters through the exact same relationship as in (3). Note that by choosing *h*(*μ*)= log(*μ*) for the geometric model, we are using a different link than the canonical link function log(*p*) implied by (7). Furthermore, for the geometric model

10 $$  FI(\mathbf{Y};\beta_{j}) = \sum\limits_{i=1}^{n} \frac{\theta(\mathbf{x}_{i})}{[1+\theta(\mathbf{x}_{i})]}x_{ij}^{2} = \sum\limits_{i=1}^{n} p(\mathbf{x}_{i})x_{ij}^{2}.  $$

Let **I**_*C*_ represent the FI matrix for ***β*** under the geometric regression model with weights *w*_*C*_(*i*)=*p*(**x**_*i*_). Then *w*_*C*_(*i*)−*w*_*B*_(*i*)=*p*(**x**_*i*_)^2^>0, and, by Result 1, the asymptotic variance of the geometric regression estimator of ***β*** is strictly less than the asymptotic variance of the logistic regression estimator. From (10) and (4), it also follows that $\sum _{i} \left [p(\mathbf {x}_{i}) x_{ij}\right ]^{2}$ represents the amount of information about *β*_*j*_ that is lost when the geometric count data **Y** are transformed into binary data **Z**.

### The Poisson model with log odds link

We now assume that the count r.v. *Y* follows the Poisson distribution with mean (and variance) *μ*:

11 $$ \Pr(Y = y) = \frac{e^{-\mu} \mu^{y}}{y!}, \quad y = 0, 1,2,\ldots.  $$

The odds of a positive count based on the Poisson distribution, *θ*_*P*_, is

12 $$ \theta_{P} = \frac{\Pr(Y > 0)}{\Pr(Y = 0)} = e^{\mu} - 1.  $$

Hence the FI in *Y* and the FI in *Z* given in (2) are related as

13 $$\begin{array}{*{20}l}  FI(Y; \theta_{P}) =& \frac{1}{\left(1 + \theta_{P}\right)^{2} \log(1 + \theta_{P})}  \\ =& \left(\frac{e^{\mu}-1}{\mu}\right) FI(Z; \theta_{P}). \end{array} $$

The last expression in (13) shows that the proportional increment in the FI in *Y*, given by $\sum _{i=1}^{\infty }\mu ^{i-1}/i!$, is substantial and increases with *μ*.

With the log odds link function, the *n* independent Poisson r.v.’s **Y**=(*Y*_1_,…,*Y*_*n*_) yield (3) with log[*θ*_*P*_(**x**_*i*_)]= log{exp[*μ*(**x**_*i*_)]−1}. Furthermore, the FI in the Poisson r.v.’s **Y** about *β*_*j*_ is given by

14 $$\begin{array}{*{20}l} FI(\mathbf{Y}; \beta_{j}) =& \sum\limits_{i=1}^{n} \frac{\left[\theta_{P}(\mathbf{x}_{i}) x_{ij}\right]^{2}}{\left[1 + \theta_{P}(\mathbf{x}_{i})\right]^{2} \log[1 + \theta_{P}(\mathbf{x}_{i})]}  \\ =& \sum\limits_{i=1}^{n} \left\{\frac{-\left[p(\mathbf{x}_{i})\right]^{2}}{\log[1 - p(\mathbf{x}_{i})]}\right\} x_{ij}^{2}  \\ =& \sum\limits_{i=1}^{n}w_{C}{(i)}x_{ij}^{2}, \end{array} $$

where *w*_*C*_(*i*) is the weight in the FI matrix for the count data. The difference in weights between the count data and the dichotomized data, *w*_*C*_(*i*)−*w*_*B*_(*i*) is

15 $$ p(\mathbf{x}_{i}) \left\{\frac{p(\mathbf{x}_{i})}{-\log[1 - p(\mathbf{x}_{i})]} - [1 - p(\mathbf{x}_{i})]\right\}.  $$

It is easily shown that for 0<*p*(**x**_*i*_)<1, the quantity within the braces above in (15) is always strictly positive and equals 0 only when *p*(**x**_*i*_)=0, but *FI*(**Y**;*β*_1_) is undefined at this value. Hence, from Result 1, it follows that the Poisson model with the log odds link function produces more efficient MLEs than the logistic model.

### The negative binomial (NB) model

The Poisson model assumes that the variance equals the mean, the geometric allows for overdispersion of the form *μ*(1+*μ*), and the NB model provides flexibility to model overdispersion with an additional parameter. When *Y* follows the NB distribution with mean parameter *μ* and dispersion parameter *δ*,

16 $$  \Pr(Y = y) = \frac{\Gamma(y + \delta)}{\Gamma(y + 1)\Gamma(\delta)}\left(\frac{\delta}{\delta + \mu}\right)^{\delta}\left(\frac{\mu}{\delta + \mu} \right)^{y}  $$

for *y*=0,1,2,…. The variance as a function of *μ* is *v*(*μ*)=*μ*(1+*μδ*^−1^). The NB distribution yields the geometric distribution when *δ*=1, and the Poisson model is obtained when *δ*→*∞*. When *Y* has NB distribution, Pr(*Y*>0)=1−[*δ*/(*δ*+*μ*)]^*δ*^, and the corresponding odds *θ* is given by

17 $$ \theta = \frac{\Pr(Y > 0)}{\Pr(Y = 0)} = \left(1 + \frac{\mu}{\delta}\right)^{\delta} - 1,  $$

and the mean as a function of *θ* is *μ*=*δ*[(1+*θ*)^1/*δ*^−1].

The second derivative of the log likelihood for a single observation *y* with respect to *θ* is

18 $$ \frac{\partial^{2} \ell}{\partial \theta^{2}} = - \left(1 + \theta\right)^{-2} \left[\frac{y}{\mu} - 1 + \frac{y}{\mu^{2}}\left(1 + \theta\right)^{1/\delta} \right]  $$

and hence the FI in the count random variable, *FI*(*Y*;*θ*), is

19 $$\begin{array}{*{20}l} & \frac{(1 + \theta)^{1/\delta}}{\mu (1 + \theta)^{2}}  \\ =& \frac{1}{\mu} \left[\left(1 + \frac{\mu}{\delta}\right)^{\delta+1} - \left(1 + \frac{\mu}{\delta}\right) \right]FI(Z; \theta). \end{array} $$

It is easy to show that when *δ*=1, *FI*(*Y*;*θ*)=*FI*(*Y*;*θ*_*G*_) (given in (9)) and as *δ*→*∞*, *FI*(*Y*;*θ*)=*FI*(*Y*;*θ*_*P*_) (given in (13)).

We now show that there is more information about *θ* in *Y* than in *Z*. From (19), it follows that, with *t*=*μ*/*δ*>0, the ratio of the two FIs can be expressed as

20 $$\begin{array}{*{20}l} & \frac{\left(1+t\right)^{\delta+1} - (1+t)}{t \delta}  \\ =& \frac{1+ (\delta+1)t +(\delta+1)\delta\left(1+t_{0}\right)^{\delta-1}\frac{t^{2}}{2!} - (1+t)}{t \delta}  \\ =& 1+ \frac{t}{2}(\delta+1)(1+t_{0})^{\delta-1}, \end{array} $$

upon using Taylor series expansion around 0. Here, *t*_0_∈(0,*t*) is positive and the second term in the last expression above represents the increase in the relative FI due to the NB fit.

The NB regression model typically uses the log link *h*(*μ*)= log(*μ*) to relate the mean of the data to the set of covariates. As noted before, we propose a regression model with log odds link function:

21 $$ \log\left[\left(1 + \frac{\mu_{i}}{\delta}\right)^{\delta} - 1\right] = \mathbf{x}_{i}^{\prime} \boldsymbol{\beta}, \quad i = 1, 2, \ldots, n.  $$

The FI in the NB r.v.’s **Y** about *β*_*j*_ is given by

22 $$ FI(\mathbf{Y}; \beta_{j}) = \sum\limits_{i=1}^{n} \frac{(1 + \theta)^{1/\delta}}{\mu (1 + \theta)^{2}} \theta^{2} x_{ij}^{2}.  $$

Now, the difference in weights between the count data and the dichotomized data, *w*_*C*_(*i*)−*w*_*B*_(*i*), is

23 $$ \frac{\theta(\mathbf{x}_{i})}{\left[1 + \theta(\mathbf{x}_{i})\right]^{2}}\left\{ \frac{\theta(\mathbf{x}_{i})\left[1 + \theta(\mathbf{x}_{i})\right]^{1/\delta}}{\mu} - 1\right\},  $$

which is strictly positive since we have shown above that the quantity inside the braces is always positive. Hence from Result 1, we conclude that the MLEs of ***β*** using the NB regression of the count data are more efficient than the MLEs using logistic regression of the dichotomized data.

The NB regression with log odds link does not fit conveniently into the GLM framework that uses iteratively weighted least squares for estimation. The model results in the following mean function

24 $$ \mu_{i} = \delta \left[\left(1 + e^{\mathbf{x}_{i}^{\prime} \boldsymbol{\beta}}\right)^{1 / \delta} - 1 \right],  $$

which is a function of both ***β*** and the dispersion parameter *δ*, and the coefficient of variation is not constant. However, our proposed NB model can be fit by maximizing the following log likelihood with respect to ***β*** and *δ*:

25 $$\begin{array}{*{20}l} \ell =& \sum\limits_{i=1}^{n} \left\{{\vphantom{\left(1 + \frac{y_{i}}{\delta}\right)}}\log\Gamma(y_{i} + \delta) - \log\Gamma(y_{i} + 1) - \log\Gamma(\delta)  \right. \\ & \quad + y_{i} \log\left\{\left[1 + \exp(\mathbf{x}_{i}^{\prime} \boldsymbol{\beta})\right]^{1/\delta} - 1 \right\}  \\ & \quad - \left.\left(1 + \frac{y_{i}}{\delta}\right)\log[1 + \exp(\mathbf{x}_{i}^{\prime} \boldsymbol{\beta})] \right\}. \end{array} $$

It is done by solving the first order conditions

26 $$\begin{array}{*{20}l} \frac{\partial \ell }{\partial \beta_{j}} =& \sum\limits_{i=1}^{n} \left\{x_{ij} \left[\frac{y_{i}}{\delta \left[1 + \exp(\mathbf{x}_{i}^{\prime} \boldsymbol{\beta})\right]^{1/\delta} - \delta} - 1 \right]\right.  \\ & \times \left.\left[\frac{\exp(\mathbf{x}_{i}^{\prime} \boldsymbol{\beta})}{1 + \exp(\mathbf{x}_{i}^{\prime} \boldsymbol{\beta})}\right] \right\} = 0, \end{array} $$

for *j*=1,2,…,*k*, and

27 $$\begin{array}{*{20}l} \frac{\partial \ell}{\partial \delta} =& \sum\limits_{i=1}^{n} \left\{{\vphantom{\frac{1^{1}}{2_{2}}}}\Psi(y_{i} + \delta) - \Psi(\delta)\right.  \\ & - \left.\frac{y_{i} \log[1 + \exp(\mathbf{x}_{i}^{\prime} \boldsymbol{\beta})]}{\delta^{2} \left[1 + \exp(\mathbf{x}_{i}^{\prime} \boldsymbol{\beta})\right]^{1/\delta} - \delta^{2}} \right\} = 0, \end{array} $$

where *Ψ*(*a*) is the first derivative of *Γ*(*a*). In the simulation studies and analyses that follow, we use the method of Nelder and Mead [16] to find MLEs of ***β*** and *δ* as implemented in the R optim function. The algorithm returns the Hessian at the maximum, which we use to estimate the standard errors of the model parameters. Starting values for the algorithm can be obtained from the coefficient estimates from the corresponding logistic regression and setting *δ*^(0)^=1.

### Simulation studies

For each distribution (geometric, Poisson, and negative binomial), we conducted a simulation study to quantify the additional precision that can be gained by using a count regression model with log odds link instead of a logistic regression model with the dichotomized data. Count data were simulated from each distribution according to the following model:

28 $$  \log(\theta_{i}) = -0.1 + 0.6x_{1i} - 0.55x_{2i} + 0.4x_{3i} + 0.25x_{4i}  $$

for *i*=1,2,…,*n*. For the NB model, *δ*=0.8. Each covariate *x*_1*i*_,…,*x*_4*i*_ in the model was a binary categorical variable. For each sample size used in the study, we simulated a (*n*×5) design matrix consisting of a column of ones and a (*n*×4) matrix of random draws from a Bernoulli(*p*=0.5) distribution. Each simulation was repeated 5000 times for sample sizes *n* = 50, 75, 100, 250, 500, and 1000. For each covariate, the OR and 95% confidence limits were calculated from the count and logistic regression models using standard methods. We compared the two approaches based on the percent bias in the estimate (measured as the percentage difference between the average of the 5000 estimates and the true OR value), the average mean squared error (MSE) for the log OR, the relative widths of the confidence intervals (measured as the average ratio of the width of the 95% confidence interval from the count regression to the width of the interval from the logistic regression), and actual coverage of the interval (measured as the percent of simulations where the confidence interval contained the true OR value obtained from the model in (28)).

In addition, we conducted a simulation study in which the data were generated using a NB distribution with dispersion parameters *δ* = 0.5, 1, 5, and 10, but the model was fit using Poisson regression with log odds. The objective of this simulation was to show how our approach would compare to logistic regression when the count distribution is misspecified.

### Analysis of real-world datasets

In addition to simulated data, we analyzed real-world datasets to assess the performance of the log odds regression model of the count data. We selected datasets for which a geometric, Poisson, or NB regression model (using log link) had already been determined to provide the best fit. We fit a regression model using the log odds link to each dataset and compared the estimates and standard errors to a logistic regression of the dichotomized data. We briefly describe each dataset below.

#### German socio-economic panel

Hilbe ([17], p. 295-297) used the geometric count model to describe the number of physician visits by *n*=2227 working women in the German Socio-Economic Panel before and after reforms to the German health system in 1997. Reforms included increases in co-payments and limits on provider reimbursement. The survey gathered data on the number of patient visits in 1996 and 1998. The data set is introduced on page 269 of Hilbe [17]. Hilbe initially fit a NB regression model, and upon identifying that the dispersion parameter *δ* was nearly one, he used the geometric model. He also compared the goodness-of-fit of the canonical link function log(1−*p*) to the log mean (or, equivalently, the log odds) link function and concluded that the latter provided a better fit (p. 297).

#### Australian health survey

Cameron and Trivedi ([15], p.77-80) fit a Poisson model to the number of doctor visits in the past two weeks reported by 5190 single adult respondents to the 1977-1978 Australian Health Survey. They used the canonical link function (that is, log mean) to model the data. In fitting a model using the log odds link function, we removed two covariates (age^2^ and presence of a chronic condition that does not limit activity) that were statistically significant in the log mean model but were not statistically significant in either the log odds model or the logistic regression model of the dichotomized data.

#### General social survey

Agresti ([18], p. 554-555) fit a NB model to count data from the 1990 General Social Survey. The survey asked 1308 participants how many people he/she knew personally that were victims of homicide in the past 12 months. Responses ranged from zero to six. The estimated model included an intercept and a single categorical covariate for race:

29 $$ \log(\hat{\mu}_{t}) = -2.3832 + 1.7331x_{t},  $$

where $\hat {\mu }_{t}$ is the estimated mean for a respondent of race category *t* and *x*_*t*_ is an indicator for that respondent’s race (1 = African-American, 0 = white). From the fitted model, Agresti estimated the ratio of means as exp(1.7331)=5.7. We fit the NB model with log odds link to estimate the OR of knowing at least one homicide victim for African-American versus white respondents. For the dichotomized data we applied the well-known asymptotic standard error for the OR calculated from a 2×2 contingency table ([18], p. 70), since our covariate of interest is binary.

## Results

### Simulation studies

Detailed results from the simulation studies are shown in Additional file 1 to this article.

The percent bias is smaller for the geometric model than the logistic regression of the dichotomized data, and the percent bias decreases as the sample size increases (Additional file 1: Table S.1). In terms of the total error (bias and variance), the MSE of the log OR for the geometric model was 29–44% of the MSE for the logistic regression model (Additional file 1: Table S.2). The relative MSE was lower (geometric model more accurate) for smaller sample sizes than for larger sample sizes. As proven in our analytic results, the geometric model consistently produced narrower confidence intervals than the logistic regression of the dichotomized data. Confidence intervals calculated directly from the count data were 56–65% as wide as the intervals from the dichotomized data. The actual coverage of the intervals from the geometric model were slightly below the nominal level of 95% for smaller sample sizes, but never less than 92.7% (*n*=50). For sample sizes of 250 or greater, actual coverage was very close to the nominal level.

Additional file 1: Table S.5, shows the percent bias in the Poisson and logistic regression estimators for each of the four covariates. For both methods, the percent bias decreases as the sample size increases, and the Poisson model produces less biased estimates. The MSE of the log OR for the Poisson approach was 52–66% of the MSE for the logistic regression approach, with lower relative MSEs for smaller sample sizes and higher relative MSEs for larger samples sizes (see Additional file 1: Table S.6). Additional file 1: Table S.7 provides the average ratio of the width of the Poisson confidence interval to the width of the logistic regression confidence interval over the 5000 simulations. In all cases, the Poisson model produced narrower intervals with relative widths in the range of 75–79%. Our assessment of the actual coverage of the nominal 95% confidence intervals for the OR showed that the coverage rates were comparable for all sample sizes and were between 94.1-95.7% (Additional file 1: Table S.8).

Additional file 1: Table S.9, indicates that the NB model with log odds link produces slightly more biased estimates than the logistic regression model of the dichotomized data. The difference is larger for smaller sample sizes, but diminishes as the sample size increases. In all cases when *n*≥75, the percent bias does not differ between the two models by more than 1.1 percentage points. Although the NB model produced slightly more biased estimates in many of the simulation scenarios considered, this bias is offset by the lower variance of the NB estimates. Additional file 1: Table S.10 shows the relative MSE of the log OR estimates. For all sample sizes and all parameters, the estimates from the NB model have a MSE that is at most half of the MSE of estimates from the logistic regression model. As an extreme example, for the *x*_2_ covariate with sample size *n*=50, the relative MSE is as low as 37%. Additional file 1: Table S.11, shows the relative width of those intervals averaged over the 5000 simulations. The intervals estimated from the NB model were, on average, shorter by 31–39% of the intervals estimated from the logistic regression model. Both methods produce confidence intervals with coverage that is very close to the nominal 95% level (Additional file 1: Table S.12).

We fit the Poisson regression model with log odds link and the logistic regression model to data that were generated with a NB distribution using dispersion parameter values of *δ* = 0.5, 1, 5 and 10. The percent bias is shown in Additional file 1: Table S.13. In these simulations, the count model with log odds link produced severely biased estimates for the model parameters when there was substantial overdispersion in the data (*δ* below 5). In some cases, the bias is so severe that the estimates have a sign that is opposite from the true parameter. In contrast, the logistic regression approach produced estimates with very low bias regardless of how much dispersion there was in the count data. As *δ* increases to 10, the data become more Poisson-like and the bias is comparable to that from the logistic regression model for small sample sizes.

### Analysis of real-world datasets

#### Geometric

Table 1 shows the OR estimates and confidence intervals estimated from both models along with the relative width of the confidence intervals, defined as the width of the interval estimated from the geometric model as a percentage of the width of the interval estimated from the logistic model. The table shows that the width of the interval from the geometric model is between 36% and 64% of the width of the interval from the logistic regression model. It also shows that there can be substantial differences in the inferences. For example, the geometric model indicates that women in the oldest age category (50–60 years) had a significantly higher odds of at least one physician visit (OR = 1.21; p = 0.009) compared to younger women (age 20–39 years). This rather intuitive result was not found in the logistic regression model (OR = 0.99; p = 0.942) because of the lower point estimate and larger standard error produced by that model.

**Table 1:** OR estimates and 95% confidence intervals from geometric and logistic regression models of physician visits for the German Socio-Economic Panel data

	Geometric		Logistic		Relative width^e^
Covariate	Estimate	Interval	Estimate	Interval	
Post-reform^a^	0.87	(0.79, 0.96)	0.82	(0.68, 0.99)	57%
Bad health^b^	3.13	(2.71, 3.63)	3.28	(2.24, 4.82)	36%
Education (10.5 - 12 years)^c^	1.09	(0.95, 1.24)	1.19	(0.94, 1.51)	50%
Education (HS graduate +)^c^	0.97	(0.84, 1.11)	1.32	(1.03, 1.70)	40%
Age (40 - 49 years)^d^	1.05	(0.93, 1.19)	0.92	(0.73, 1.16)	62%
Age (50 - 60 years)^d^	1.21	(1.05, 1.39)	0.99	(0.76, 1.29)	64%
Log household income	1.13	(0.99, 1.30)	1.26	(0.98, 1.62)	48%

Reference category: ^a^ Pre-reform; ^b^ Good health; ^c^ 7 - 10 years of education; ^d^ Age 20–39 years ^e^ Geometric compared to logistic model

Although the interpretation of the parameters is the same under both geometric and logistic regression, each model is a different method of estimation for those parameters. Both approaches produce asymptotically unbiased estimators as they are based on the method of maximum likelihood, but each may be biased for small sample sizes. We compared their small sample bias over a range of sample sizes using the coefficient estimates from the fitted geometric model, given in Table 1 as the true parameter values. We simulated 1000 samples of different sizes ranging from 50 to 500 and applied each method to to obtain the MLEs of the ORs. We then calculated the percent bias, defined as the difference between the average of the 1000 estimates and the true value as a percent of the true value. The results are shown in Additional file 1: Figure S.1. The geometric model almost always has smaller bias that is close to zero, even when there are fewer than 100 observations. The conclusion is that the geometric model produces substantially more accurate and precise estimators than the logistic model when the counts arise from a geometric distribution.

#### Poisson

Table 2 shows the parameter estimates and 95% confidence intervals for the Australian Health Survey data. It also reports the ratio of the widths of the intervals from the Poisson model and the logistic regression model. For all of the covariates, the Poisson model produces narrower intervals with relative widths that ranged from 68%–90%. The statistical inference differs across the two models in two cases (regarding health insurance coverage status). In both of these cases, the difference arises because the logistic regression produces substantially higher point estimates than the Poisson regression with log odds link (1.30 versus 1.17 for private insurance, 1.53 versus 1.20 for free government insurance due to old age, disability, or veteran status), resulting in statistical significance with the former and nonsignificance with the latter model. For the free government insurance coefficient, the Poisson model has the largest reduction in variance over the logistic regression model (average relative width of 68%), but the large difference in point estimates offsets these gains to produce different statistical inferences.

**Table 2:** OR estimates and 95% confidence intervals from Poisson and logistic regression models of physician visits for the Australian Health Survey data

	Poisson		Logistic		Relative width^d^
Covariate	Estimate	Interval	Estimate	Interval	
Female	1.28	(1.11, 1.47)	1.30	(1.11, 1.53)	85.85%
Age^a^	1.67	(1.10, 2.52)	1.71	(1.06, 2.74)	84.71%
Income^b^	0.82	(0.66, 1.01)	0.95	(0.75, 1.20)	76.49%
Private insurance^c^	1.17	(0.98, 1.39)	1.30	(1.07, 1.59)	78.51%
Free government insurance (low income)^c^	0.55	(0.36, 0.85)	0.50	(0.30, 0.84)	89.64%
Free government insurance (old age, disability, veteran)^c^	1.20	(0.95, 1.53)	1.53	(1.16, 2.01)	68.28%
Number of illnesses in past two weeks	1.30	(1.24, 1.36)	1.32	(1.25, 1.39)	85.36%
Number of days of reduced activity in past two weeks	1.24	(1.21, 1.26)	1.17	(1.14, 1.20)	78.17%
General health questionnaire score	1.05	(1.02, 1.08)	1.06	(1.03, 1.10)	83.68%
Has chronic condition that limits activity	1.17	(0.98, 1.41)	1.19	(0.96, 1.48)	84.20%

^a^ Age in years divided by 100 ^b^ Annual income in tens of thousands of dollars ^c^ Reference category: government Medibank insurance ^d^ Poisson compared to logistic model

#### Negative binomial

We applied the NB model with log odds link to estimate the OR of knowing at least one homicide victim for African-American versus white respondents. The fitted model yielded the following MLEs:

30 $$\begin{array}{*{20}l} & \log\left[\hat{\theta}{(t)}\right] = -2.5387 + 1.3154x_{t}  \\ & \hat{\delta} = 0.2023  \\ & \hat{OR}_{NB} = \exp(1.3154) = 3.726. \end{array} $$

An approximate 95% confidence interval for log(*OR*) is (0.9635,1.6674), which when exponentiated yields an interval of (2.62,5.30) for the OR. Thus we conclude that African-American respondents had a higher odds of knowing at least one homicide victim compared to white respondents. Note that there is non-zero covariance between the estimator of the regression coefficients and the estimator of the dispersion parameter, unlike the NB model with log mean link ([15], p.82).

From the dichotomized data, we obtain $\hat {OR}_{B} = 4.553$ and $SE \left [\log \left (\hat {OR}_{B} \right) \right ] = 0.217$, that leads us to an approximate 95% confidence interval (2.98,6.96) for the OR. In this situation, our conclusion remains the same even though the NB model produced a lower estimate than the dichotomized data model (3.7 versus 4.6) and an interval that was only 83% as wide.

## Discussion

**Fig. 1:**
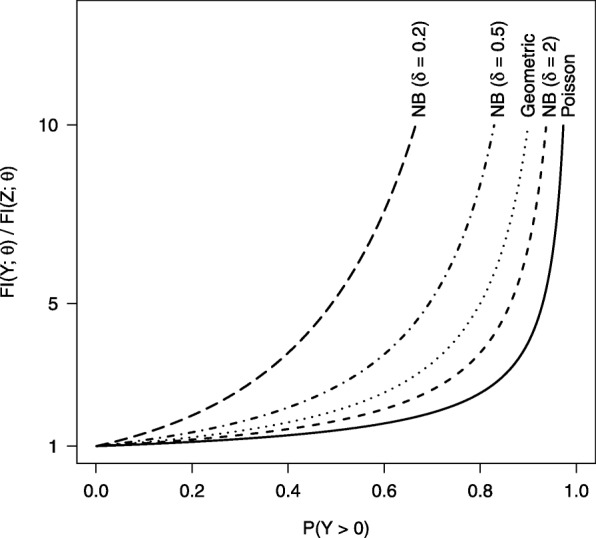
Comparison of information gains across count models. The figure shows *FI*(*Y*;*θ*)/*FI*(*Z*;*θ*) as a function of the *p*= Pr(*Y*>0) for various count models. As the probability of a positive count increases, the relative FI increases. The gains are largest for models with the most dispersion in the counts

Figure 1 shows the relative Fisher information *FI*(*Y*;*θ*)/*FI*(*Z*;*θ*) as a function of *p*= Pr(*Y*>0) for the count models considered in this study. The information lost from dichotomization is modest for small *p*, but grows exponentially as *p*→1. The rate at which information is lost from dichotomization is directly related to the amount of overdispersion in the data. Under the Poisson model, which assumes no overdispersion, the rate of increase in *FI*(*Y*;*θ*)/*FI*(*Z*;*θ*) becomes large near *p*=0.8. Conversely, under the NB model with *δ*=0.2, *FI*(*Y*;*θ*)/*FI*(*Z*;*θ*) begins to increase significantly near *p*=0.3. Dichotomization of count data loses all information regarding overdispersion in the data, information that is critical for accurate estimation of OR.

Analyzing data with a count regression model can lead to different statistical inferences compared to a logistic regression analysis of the dichotomized counts, as we showed through two examples with data sets from the literature. In our examples, statistical significance differed between the two approaches not so much because of differences in standard errors, but because the logistic regression model tended to provide larger point estimates for some model parameters. Our simulations suggest that logistic regression using dichotomized data tends to produce a larger positive bias than count regression models for the geometric and Poisson distributions. Thus, logistic regression may identify more statistically significant parameter estimates, but these conclusions may be inaccurate due to bias in the estimation procedure.

Traditionally, the Poisson and NB regression models have employed the log link function for the mean (*μ*), while we have used the log link function for the odds. The log(*μ*) link function is usually appropriate when the objective of the analysis is to compare means between two groups. As noted in the “Background” section, some researchers prefer instead to compare the odds of a positive count between two groups. For these types of analyses, we recommend the use of the log odds link function, and it facilitates a direct comparison with the logistic regression model where the regression coefficients carry the same interpretation in terms of OR. Luckily for the geometric model, both link functions match. One could consider the traditional modeling approach for the Poisson and NB models using the log link function for *μ*. However, in that case, the log odds cannot be expressed as a linear function of the regression coefficients involved and a direct comparison with the logistic regression model will not be possible. When analyzing count data, therefore, the analyst must first decide which parameters to compare (means or odds), then choose the link function accordingly.

The incompatibility of the log(*μ*) and log odds (for 0 counts) link functions has been discussed by Heilbron [19] for the Poisson and NB models. In order to make the link functions compatible, he has suggested the transformation *P*(*μ*)= Pr(*Y*>0), with the corresponding log mean link function expressed as *h*(*P*)= log(*μ*)= log[*P*^−1^(*μ*)]. For example, for the Poisson parent, *P*(*μ*)=1− exp(−*μ*) and

31 $$ h(P) = \log\{-\log[1 - \Pr(Y > 0 | \mathbf{x})]\} = \mathbf{x}^{\prime} \boldsymbol{\beta}  $$

would make the two link functions compatible (as shown in his Table 2). As noted before, for the geometric model, incompatibility does not arise, and to ensure compatibility for the NB model, one needs to assume that log(*μ*(***x***)) is of the form **x**^′^***β***, with the constraint that *μ*=*δ*[(1+*θ*)^1/*δ*^−1] (see (17)). Thus, in Heilbron’s approach, log(*θ*) cannot be linear in the components of **x**. In contrast, the link function we choose for *μ* is defined through the properties of log(*θ*); for example, for the Poisson model, we assume log{exp[*μ*(**x**)−1]}=**x**^′^***β***.

The above incompatibility can be handled in a zero-altered, two-part hurdle model where relevant parameters are assumed to have a simple relationship. This approach was initially considered by Heilbron [19], and more recently by Preisser et al. [10]. Their models introduce a new parameter for the probability of a zero count (and hence for the odds for a positive count) and link it to the mean of the distribution modeling the positive counts. Heilbron [19] and Preisser et al. [10] discuss the implications of the compatibility assumption that is needed for the inference presented. In any case, it does not work for the commonly used Poisson and NB models that do not contain excess zero counts.

Whatever model one settles on, a correctly fit count model is expected to produce better inference for the OR than the one produced by the logistic regression model because the dichotomized data do not produce a sufficient statistic for the count data. As with any model fitting, it is important that the analyst choose the correct distribution to match the data. Our simulation demonstrated that the use of the Poisson model can produce heavily biased estimates of the OR when there is overdispersion in the data. A logistic regression of the dichotomized counts is robust to this kind of model misspecification. The goodness-of-fit of various count models, as well as link functions, can be assessed through model diagnostics [12]. With the availability of efficient algorithms for computing the MLEs in quite general contexts, computational issues are not a major concern in this pursuit.

In this paper we have addressed implications of our model assumptions on inference through point and interval estimates using the maximum likelihood estimators. Whatever model one uses, it is known that the MLEs are functions of sufficient statistics, consistent and are asymptotically normal efficient estimators (under some standard regularity conditions). The test statistics, such as the one in the commonly used Wald’s test, contain the standardized MLE with standard error of the MLE in the denominator. A reduction in the standard error (or equivalently, increase in the Fisher information) results in increased power that can be approximated by tails of the standard normal distribution. Our results imply increased power when Wald’s test is used. Bias will affect both power and type I error, and we have not studied the implications in detail. Instead, we have chosen to focus on confidence interval lengths and coverage probabilities that illustrate these effects quite efficiently. The confidence intervals are also free of the situation-specific research hypotheses.

The OR is often of interest to biomedical researchers. For rare events, the OR, given by [*p*_1_/(1−*p*_1_)]/[*p*_2_/(1−*p*_2_)], closely approximates the relative risk (RR), *p*_1_/*p*_2_. If one is specifically interested in *p* that is not small, log(*p*) can be used as the link function. Zou [20] has provided a model for estimating RR for binary data using this link function and has used Poisson regression with robust standard errors to fit the model. Our approach can be easily modified for that link function to model RR directly from a count data to obtain more precise inference on RR than what is achievable with the dichotomized data.

## Conclusions

The OR is a commonly used measure of uncertainty in a binary decision (e.g., zero or non-zero). When data are obtained from a count process, there is information in the counts that is lost when the data are dichotomized. We proposed a method for estimating the OR that does not require dichotomizing the count data. We demonstrated analytically the gain in information using this approach and the resulting increase in precision when making inferences on the OR. For a given *p*, the probability of a positive count, the information gain increases as the count data become more dispersed from zero and one. The analytic methods we propose can be implemented easily by biomedical researchers. For geometric and Poisson models, any software fitting GLMs can be used provided it allows the user to modify the link function. For NB models, an optimization routine can be used to maximize the likelihood. In our examples, the optimization always converged because the logistic regression of the dichtomized data provides initial values that are very close to the solutions for the count data.

## Additional file

Additional File 1 Additional File 1 is a PDF file with detailed results from the simulation studies. (PDF 86 kb)

## Abbreviations

ARE: Asymptotic relative efficiency
FI: Fisher information
GLM: Generalized linear model
MLE: Maximum likelihood estimate
MSE: Mean squared error
NB: Negative binomial
OR: Odds ratio
RR: Relative risk
r.v.: Random variable
VA: Veterans Administration
