# Supplementary material for: Odds ratios from logistic, geometric, Poisson, and negative binomial regression models
Source: BMC Med Res Methodol. 2018 Oct 20;18:112. doi: 10.1186/s12874-018-0568-9 (PMC6195979; doi:10.1186/s12874-018-0568-9)
Supplement: Supplementary file 1 — Additional File 1 is a PDF file with detailed results from the simulation studies. (PDF 86 kb) [file 12874_2018_568_MOESM1_ESM.pdf]

Additional File 1

Detailed Simulation Results from Odds Ratios from Logistic, Geometric, Poisson, and Negative Binomial Regression Models

Christopher J. Sroka and Haikady N. Nagaraja

Table S.1: Percent Bias in OR Estimates from Geometric (G) and Logistic Regression (LR) Models

	True		Percent bias for various sample sizes (n)					
Covariate	OR	Model	$n = 1000$	$n = 500$	$n = 250$	$n = 100$	$n = 75$	$n = 50$
x_1	1.822	G	0.1%	-0.4%	-0.2%	-0.1%	1.1%	1.5%
		LR	0.4%	0.1%	0.7%	2.7%	3.7%	6.3%
x_2	0.577	G	-0.2%	-0.2%	-0.5%	-0.1%	0.1%	-0.5%
		LR	-0.6%	-0.6%	-0.7%	-2.9%	-3.4%	-7.5%
x_3	1.492	G	-0.1%	0.1%	0.4%	-0.5%	0.7%	0.1%
		LR	0.2%	0.3%	1.2%	2.4%	4.1%	7.1%
x_4	1.284	G	-0.1%	0.2%	0.3%	-0.3%	0.2%	1.6%
		LR	-0.2%	0.6%	0.6%	1.5%	2.3%	3.0%

Table S.2: Relative Mean Squared Error (MSE)[a] of log OR from Geometric and Logistic Regression Models

	True	Sample sizes (n)					
Covariate	OR	$n = 1000$	$n = 500$	$n = 250$	$n = 100$	$n = 75$	$n = 50$
x_1	1.822	43.6%	41.4%	39.2%	40.9%	40.8%	39.8%
x_2	0.577	42.0%	40.3%	41.0%	36.5%	37.3%	29.4%
x_3	1.492	42.0%	41.4%	38.3%	38.3%	37.0%	29.4%
x_4	1.284	42.0%	41.3%	39.6%	37.6%	35.3%	36.6%

[a]MSE for Poisson model as a percentage of MSE for logistic regression

Table S.3: Average Relative Width of Confidence Interval[a] for OR from Geometric and Logistic Regression Models

Covariate	True OR	Sample sizes (n) $n = 1000$	$n = 500$	$n = 250$	$n = 100$	$n = 75$	$n = 50$
x_1	1.822	64.6%	64.0%	63.1%	60.9%	60.7%	57.7%
x_2	0.577	64.7%	64.5%	64.0%	62.2%	60.5%	59.5%
x_3	1.492	64.4%	63.9%	62.0%	59.4%	57.9%	56.0%
x_4	1.284	64.7%	64.0%	62.2%	59.8%	57.5%	58.4%

[a]The width of the 95% confidence interval under the Geometric model divided by the width under the logistic regression model averaged across all simulations

Table S.4: Actual Coverage of Nominal 95% Confidence Interval from Geometric (G) and Logistic Regression (LR) Models

Covariate	True OR	Model	Actual coverage for various sample sizes (n) $n = 1000$	$n = 500$	$n = 250$	$n = 100$	$n = 75$	$n = 50$
x_1	1.822	G	94.7%	94.7%	94.8%	93.8%	93.7%	92.7%
		LR	95.2%	94.9%	94.4%	94.5%	95.4%	94.9%
x_2	0.577	G	95.0%	95.4%	94.9%	94.4%	93.3%	92.9%
		LR	94.8%	94.9%	94.9%	94.6%	94.6%	94.5%
x_3	1.492	G	94.8%	94.6%	94.6%	93.8%	93.4%	93.7%
		LR	95.1%	94.9%	95.0%	94.7%	94.3%	94.6%
x_4	1.284	G	95.1%	95.1%	94.7%	94.0%	94.1%	93.1%
		LR	95.4%	94.8%	94.8%	94.2%	94.9%	95.0%

Table S.5: Percent Bias in OR Estimates from Poisson (P) and Logistic Regression (LR) Models

Covariate	True OR	Model	Percent bias for various sample sizes (n) $n = 1000$	$n = 500$	$n = 250$	$n = 100$	$n = 75$	$n = 50$
x_1	1.822	P	0.4%	0.4%	1.3%	0.5%	2.0%	3.2%
		LR	0.5%	0.7%	2.0%	2.7%	4.2%	7.2%
x_2	0.577	P	0.0%	0.3%	-1.0%	0.0%	-2.4%	-3.0%
		LR	-0.2%	0.0%	-1.4%	-2.3%	-5.4%	-7.5%
x_3	1.492	P	0.0%	0.2%	0.6%	0.5%	0.5%	1.2%
		LR	0.3%	0.5%	1.0%	2.5%	2.7%	3.8%
x_4	1.284	P	0.1%	0.0%	0.4%	-1.2%	0.2%	1.9%
		LR	0.2%	0.2%	0.8%	1.0%	1.9%	2.0%

Table S.6: Relative Mean Squared Error (MSE)[a] of log OR from Poisson and Logistic Regression Models

Covariate	True OR	Sample sizes (n) $n = 1000$	$n = 500$	$n = 250$	$n = 100$	$n = 75$	$n = 50$
x_1	1.822	65.8%	60.7%	63.1%	58.7%	59.6%	60.8%
x_2	0.577	63.2%	62.4%	61.9%	58.6%	55.1%	52.0%
x_3	1.492	62.8%	63.2%	60.4%	55.6%	56.0%	55.1%
x_4	1.284	61.8%	62.9%	59.2%	58.8%	55.3%	53.6%

[a]MSE for Poisson model as a percentage of MSE for logistic regression

Table S.7: Average Relative Width of Confidence Interval[a] for OR from Poisson and Logistic Regression Models

Covariate	True OR	Sample sizes (n) $n = 1000$	$n = 500$	$n = 250$	$n = 100$	$n = 75$	$n = 50$
x_1	1.822	79.2%	78.8%	78.0%	76.4%	76.3%	74.5%
x_2	0.577	79.3%	79.2%	79.1%	78.4%	77.4%	76.1%
x_3	1.492	79.1%	78.8%	77.6%	75.7%	74.8%	74.5%
x_4	1.284	79.3%	79.1%	77.7%	75.5%	74.2%	76.2%

[a]The width of the 95% confidence interval under the Poisson model divided by the width under the logistic regression model averaged across all simulations

Table S.8: Actual Coverage of Nominal 95% Confidence Interval from Poisson (P) and Logistic Regression (LR) Models

Covariate	True OR	Model	Actual coverage for various sample sizes (n) $n = 1000$	$n = 500$	$n = 250$	$n = 100$	$n = 75$	$n = 50$
x_1	1.822	P	95.0%	94.8%	94.8%	94.8%	94.8%	95.7%
		LR	95.7%	94.6%	95.2%	94.8%	94.6%	94.9%
x_2	0.577	P	94.2%	94.9%	95.0%	94.8%	94.8%	94.8%
		LR	94.4%	95.2%	94.9%	95.0%	94.3%	94.6%
x_3	1.492	P	94.1%	95.0%	95.1%	94.8%	95.6%	94.6%
		LR	94.6%	95.2%	95.7%	94.5%	95.1%	94.6%
x_4	1.284	P	95.0%	94.4%	94.9%	94.7%	94.6%	95.2%
		LR	94.8%	94.6%	94.6%	95.3%	95.0%	94.8%

Table S.9: Relative Bias in OR Estimates from Negative Binomial (NB) and Logistic Regression (LR) Models

Covariate	True OR	Model	Percent bias for various sample sizes (n) $n = 1000$	$n = 500$	$n = 250$	$n = 100$	$n = 75$	$n = 50$
x_1	1.822	NB	0.4%	0.9%	1.6%	5.2%	5.9%	10.2%
		LR	0.4%	0.6%	1.7%	4.5%	4.9%	6.7%
x_2	0.577	NB	-0.3%	-0.7%	-1.6%	-3.8%	-4.7%	-5.9%
		LR	-0.4%	-0.7%	-0.9%	-3.9%	-4.0%	-6.9%
x_3	1.492	NB	0.2%	0.6%	1.0%	2.1%	4.3%	5.5%
		LR	0.0%	0.6%	0.5%	1.9%	3.2%	5.5%
x_4	1.284	NB	0.1%	0.4%	0.8%	0.1%	1.7%	5.7%
		LR	-0.1%	0.3%	0.6%	0.3%	1.4%	3.3%

Table S.10: Relative Mean Squared Error (MSE)[a] of log OR from Negative Binomial and Logistic Regression Models

Covariate	True OR	Sample sizes (n) $n = 1000$	$n = 500$	$n = 250$	$n = 100$	$n = 75$	$n = 50$
x_1	1.822	42.1%	42.3%	42.3%	45.8%	47.5%	50.9%
x_2	0.577	40.6%	43.0%	43.1%	43.1%	42.1%	37.4%
x_3	1.492	40.2%	40.9%	39.5%	41.9%	42.3%	44.8%
x_4	1.284	41.1%	39.7%	38.4%	39.6%	39.7%	43.8%

[a]MSE for negative binomial model as a percentage of MSE for logistic regression

Table S.11: Average Relative Width of Confidence Interval[a] for OR from Negative Binomial and Logistic Regression Models

Covariate	True OR	Sample sizes (n) $n = 1000$	$n = 500$	$n = 250$	$n = 100$	$n = 75$	$n = 50$
x_1	1.822	64.3%	64.4%	64.1%	65.8%	66.4%	69.1%
x_2	0.577	63.7%	63.7%	63.5%	63.1%	61.9%	62.4%
x_3	1.492	63.1%	62.8%	62.2%	63.0%	63.1%	64.6%
x_4	1.284	62.6%	62.6%	61.4%	62.1%	60.9%	65.6%

[a]The width of the 95% confidence interval under the negative binomial model divided by the width under the logistic regression model averaged across all simulations

Table S.12: Actual Coverage of Nominal 95% Confidence Interval from Negative Binomial (NB) and Logistic Regression (LR) Models

Covariate	True OR	Model	Actual coverage for various sample sizes (n) $n = 1000$	$n = 500$	$n = 250$	$n = 100$	$n = 75$	$n = 50$
x_1	1.822	NB	95.3%	95.2%	94.7%	94.7%	94.5%	93.8%
		LR	94.9%	94.9%	95.0%	95.2%	95.0%	95.2%
x_2	0.577	NB	95.2%	94.8%	94.4%	94.3%	94.7%	94.6%
		LR	95.1%	95.1%	94.9%	94.4%	94.1%	94.7%
x_3	1.492	NB	95.3%	95.0%	95.5%	94.8%	94.9%	94.4%
		LR	95.3%	95.2%	95.7%	95.0%	94.8%	94.7%
x_4	1.284	NB	95.0%	95.0%	95.1%	94.2%	93.9%	94.0%
		LR	95.4%	95.0%	95.0%	94.5%	95.1%	94.3%

Table S.13: Relative Bias in OR Estimates from Poisson (P) and Logistic Regression (LR) Models When Data Follow a Negative Binomial Distribution

$\delta = 0.5$

Covariate	True OR	Model	Percent bias for various sample sizes (n) $n = 1000$	$n = 500$	$n = 250$	$n = 100$	$n = 75$	$n = 50$
x_1	1.822	P	411.0%	438.2%	475.9%	419.9%	412.6%	356.7%
		LR	0.2%	0.6%	0.7%	2.7%	4.4%	7.7%
x_2	0.577	P	-78.1%	-78.7%	-79.9%	-79.2%	-82.3%	-78.3%
		LR	-0.3%	-0.9%	-0.9%	-3.0%	-4.1%	-7.4%
x_3	1.492	P	196.2%	217.7%	234.4%	223.7%	252.5%	173.4%
		LR	0.0%	0.3%	0.6%	2.5%	3.7%	6.2%
x_4	1.284	P	99.4%	101.8%	115.2%	137.3%	105.7%	62.8%
		LR	0.1%	0.3%	0.2%	1.9%	1.8%	4.1%

$\delta = 1$

Covariate	True OR	Model	Percent bias for various sample sizes (n) $n = 1000$	$n = 500$	$n = 250$	$n = 100$	$n = 75$	$n = 50$
x_1	1.822	P	67.9%	69.7%	73.6%	71.2%	72.6%	70.7%
		LR	0.5%	0.5%	1.2%	3.1%	6.3%	8.7%
x_2	0.577	P	-38.2%	-38.6%	-39.4%	-39.3%	-40.5%	-40.8%
		LR	-0.6%	-0.8%	-0.9%	-3.3%	-3.7%	-10.3%
x_3	1.492	P	40.4%	43.9%	45.2%	45.2%	48.2%	42.9%
		LR	0.0%	0.7%	0.5%	1.0%	4.0%	8.1%
x_4	1.284	P	24.3%	25.6%	26.5%	27.7%	24.9%	20.4%
		LR	-0.1%	0.3%	0.0%	1.4%	1.4%	1.2%

$\delta = 5$

Covariate	True OR	Model	Percent bias for various sample sizes (n) $n = 1000$	$n = 500$	$n = 250$	$n = 100$	$n = 75$	$n = 50$
x_1	1.822	P	7.9%	8.1%	8.6%	10.5%	10.1%	10.3%
		LR	0.1%	0.7%	1.1%	4.1%	4.8%	6.1%
x_2	0.577	P	-6.8%	-6.7%	-7.7%	-7.9%	-7.6%	-9.5%
		LR	-0.2%	-0.2%	-1.4%	-3.5%	-3.9%	-8.4%
x_3	1.492	P	5.5%	5.4%	5.7%	6.1%	7.2%	7.4%
		LR	0.3%	0.4%	0.5%	3.1%	4.1%	5.6%
x_4	1.284	P	3.5%	3.3%	3.0%	4.5%	4.8%	4.9%
	LR	0.3%	0.0%	-0.1%	2.6%	3.1%	2.6%	

$\delta = 10$

Covariate	True OR	Model	Percent bias for various sample sizes (n) $n = 1000$	$n = 500$	$n = 250$	$n = 100$	$n = 75$	$n = 50$
x_1	1.822	P	3.9%	4.1%	4.5%	5.1%	5.5%	7.7%
		LR	0.3%	0.5%	1.1%	2.8%	5.1%	7.7%
x_2	0.577	P	-3.5%	-3.3%	-3.7%	-4.3%	-5.1%	-6.4%
		LR	-0.3%	-0.2%	-1.0%	-3.4%	-4.8%	-9.7%
x_3	1.492	P	2.4%	2.6%	3.3%	2.5%	4.2%	4.1%
		LR	0.1%	0.3%	1.3%	1.9%	3.5%	6.3%
x_4	1.284	P	1.7%	1.8%	1.9%	1.8%	1.3%	3.8%
		LR	0.1%	0.0%	0.2%	1.2%	1.9%	1.3%

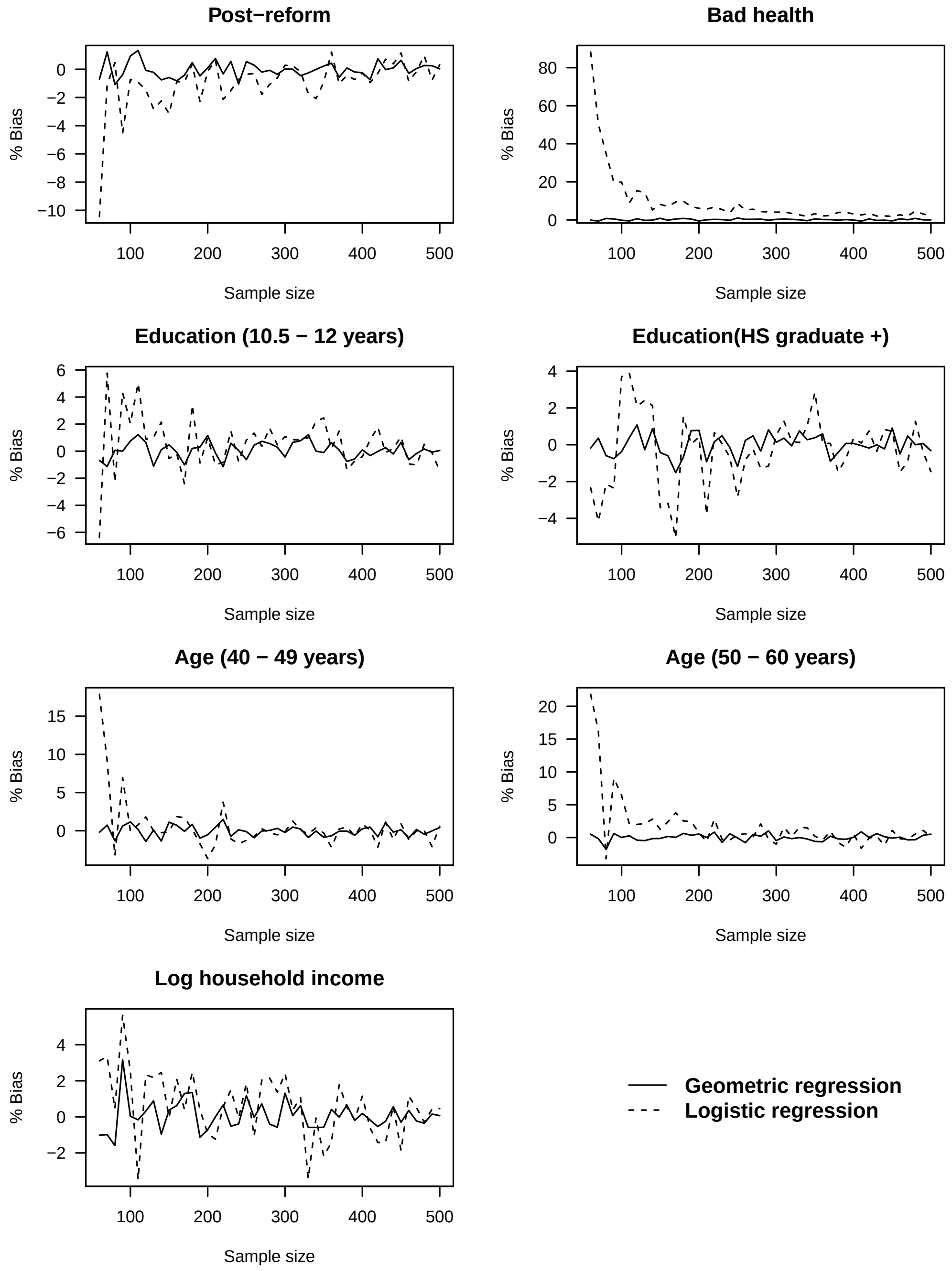

Figure S.1: Percent bias associated with ORs estimated from geometric and logistic regressions of physician visits
